# Editorial Department

**Published:** 1856-02

**Authors:** 


The October (1855) number of the Medico-Chirurgical Review, has a review of the works of Drs. Jenner and Flint, on the non-identity of typhus and typhoid fevers. At the close of the article, the writer, Dr. Begbie, pays the following high compliment to the three *nomina digna*, Louis, Jenner and Flint:

"It must not be forgotten that the authors of three countries—of France and America, besides our own—have added imperishable monuments to their talents and labors in the cultivation of this most interesting subject. Of the researches of Dr. Flint, the latest American observer, as embodied in the work we have had under our notice, we are very glad to express a high opinion. Not free from confusion in some particulars, from causes its author indicates in his preface, Dr. Flint's reports contains a body of most useful facts, and for the most part very accurate deductions from these facts, which are equally honorable for Dr. Flint and to the medical school of which he is so distinguished a teacher."

This hearty recognition of the labors of our friend on the part of the foremost medical quarterly in the world, is a pleasant, though so far as our own opinions are concerned, unnecessary endorsement of the value of his researches. We feel a pride in the fact that they were first published in the Buffalo Medical Journal, as well as that it is to be still favored by contributions from his pen. The series of clinical lectures on diseases of the skin, already commenced in our pages, by him, will be continued during the present year.

